# Investigation of the rimegepant effect on cerebral and extracerebral arteries during migraine attacks: a longitudinal magnetic resonance angiography study

**DOI:** 10.1093/braincomms/fcag004

**Published:** 2026-01-09

**Authors:** Basit Ali Chaudhry, Samaira Younis, Hassan Al-Mashat, Emil Gozalov, Tariq Mohammad Amin, Patrick J H de Koning, Henrik Bo Wiberg Larsson, Faisal Mohammad Amin

**Affiliations:** Department of Neurology, Danish Headache Center, Copenhagen University Hospital—Rigshospitalet, Glostrup, DK- 2600 Glostrup, Denmark; Department of Clinical Medicine, Faculty of Health and Medical Sciences, University of Copenhagen, Copenhagen, DK- 2200 Glostrup, Denmark; Department of Neurology, Danish Headache Center, Copenhagen University Hospital—Rigshospitalet, Glostrup, DK- 2600 Glostrup, Denmark; Department of Neurology, Danish Headache Center, Copenhagen University Hospital—Rigshospitalet, Glostrup, DK- 2600 Glostrup, Denmark; Department of Neurology, Danish Headache Center, Copenhagen University Hospital—Rigshospitalet, Glostrup, DK- 2600 Glostrup, Denmark; Department of Neurology, Danish Headache Center, Copenhagen University Hospital—Rigshospitalet, Glostrup, DK- 2600 Glostrup, Denmark; Division of Image Processing, Department of Radiology, Leiden University Medical Center, Leiden, 2300 RC Leiden, Netherlands; Department of Clinical Medicine, Faculty of Health and Medical Sciences, University of Copenhagen, Copenhagen, DK- 2200 Glostrup, Denmark; Functional Imaging Unit, Department of Clinical Physiology and Nuclear Medicine, Copenhagen University Hospital Rigshospitalet-Glostrup, Glostrup, DK- 2600 Glostrup, Denmark; Department of Neurology, Danish Headache Center, Copenhagen University Hospital—Rigshospitalet, Glostrup, DK- 2600 Glostrup, Denmark; Department of Clinical Medicine, Faculty of Health and Medical Sciences, University of Copenhagen, Copenhagen, DK- 2200 Glostrup, Denmark

**Keywords:** migraine without aura, menstrual migraine, meningeal arteries, CGRP, gepants

## Abstract

Migraine is a leading cause of disability worldwide, and triptans, the most widely used acute treatment, act through vasoconstriction and are contraindicated in patients with vascular disease. Rimegepant, a calcitonin gene-related peptide receptor antagonist, is proposed as a non-vasoconstrictive alternative, but its direct vascular effects during spontaneous migraine attacks have not been examined. This was a prospective, longitudinal study conducted at a single academic imaging centre between 12 January 2024 and 10 June 2025. Eighteen women aged 18–40 years with menstrually related migraine without aura were enrolled. Fifteen participants completed high-resolution 3 T magnetic resonance angiography during a spontaneous migraine attack before, and at 30 and 60 min after, administration of a single 75 mg oral dose of rimegepant. The primary outcome was change in arterial circumference of the cerebral artery and meningeal artery. Circumference was measured in millimetre and compared using paired samples *t*-tests. No significant vasoconstriction was observed in either artery following rimegepant administration. Cerebral artery circumference remained stable (baseline 8.13 ± 0.93 mm; 30 min 8.02 ± 0.84 mm, *P* = 0.404; 60 min 8.15 ± 0.90 mm, *P* = 0.918). Meningeal artery circumference showed no significant change (baseline 4.30 ± 0.83 mm; 30 min 4.47 ± 0.68 mm, *P* = 0.084; 60 min 4.35 ± 0.78 mm, *P* = 0.688). Rimegepant did not induce measurable constriction of cerebral or meningeal arteries during spontaneous migraine attacks. These findings support its vascular safety and indicate that effective migraine relief with calcitonin gene-related peptide receptor antagonists does not depend on vasoconstriction, in contrast to triptan therapy.

## Teaser

Chaudhry *et al*. investigated the vascular effects of rimegepant during spontaneous migraine attacks using magnetic resonance angiography. The study showed that rimegepant did not constrict cerebral or meningeal arteries, supporting its vascular safety and demonstrating that effective migraine treatment can occur without arterial constriction

## Graphical Abstract

**Graphical Abstract:**
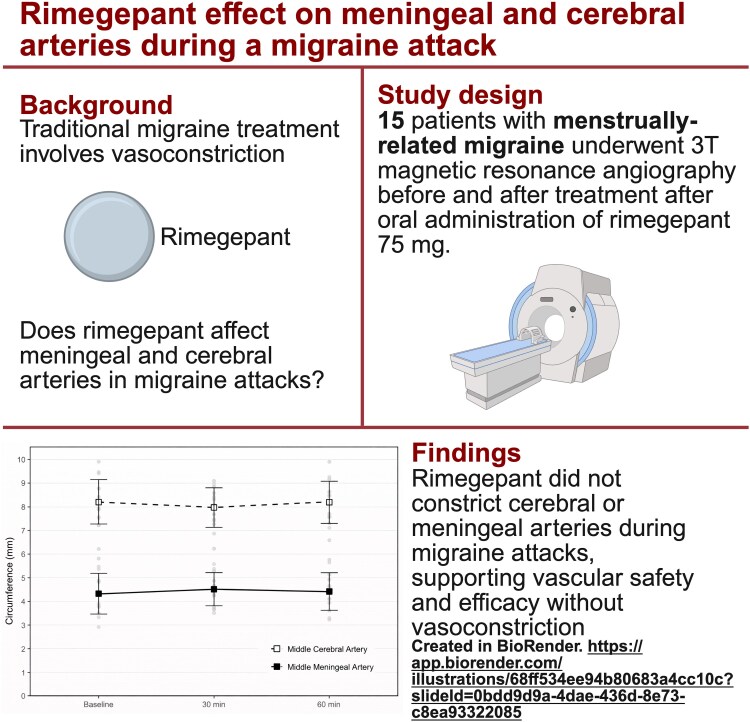

## Introduction

Migraine is a highly prevalent neurological disorder and a leading cause of disability worldwide, particularly among women of reproductive age.^1^ It affects ∼15% of the adult population^2,3^ and is often associated with significant personal and socioeconomic burden.^4^ Migraine attacks can be severe, prolonged and sometimes refractory to conventional acute treatments.^5^

The calcitonin gene-related peptide (CGRP) antagonist rimegepant is a relatively novel antimigraine medication approved for both acute and preventive treatments.^6,7^ Rimegepant has demonstrated good efficacy and a favourable safety profile in randomized controlled trials.^7,8^ Preclinical studies have reported no direct vascular effects of CGRP antagonists, positioning gepants as non-vascular alternatives to triptans.^9,10^ On the contrary, it has been suggested that CGRP antagonists may have indirect vasoconstrictor effects, via inhibition of the CGRP-induced vasodilation.^11,12^ In clinical practice, this has led to precautions regarding the use of gepants in patients with a history of cerebrovascular event or those at increased risk.

We have previously shown that sumatriptan has a selective vasoconstrictor effect on the meningeal arteries using high-resolution magnetic resonance angiography (MRA) in healthy subjects as well as women with ongoing migraine attacks.^13,14^ Studies using MRA in individuals with migraine attacks have consistently found that sumatriptan only induces vasocontraction of extracerebral arteries, such as MMA, and no vascular effects on intracerebral arteries.^14-16^ Moreover, it has been discussed whether arterial constriction is necessary for the acute antimigraine effect as lasmiditan induces its antimigraine effects via a non-vascular, primarily neural, mechanism.^17^

To the best of our knowledge, the vascular effects of rimegepant or other gepants have never been investigated *in vivo* in individuals with migraine. In the present study, we used MRA to measure circumference of meningeal and cerebral arteries before and after oral administration of rimegepant *in vivo* during spontaneous attacks of migraine.

## Materials and methods

### Recruitment, inclusion and exclusion criteria

All participants were recruited via an announcement on a Danish website for recruitment of participants with migraine. All participants were eligible if they had regular menstrual cycle (>12 months), age between 18 and 40 years and a diagnosis of menstrually related migraine without aura according to the International Classification of Headache Disorders (ICHD-3).^18^ Neuroimaging of spontaneous attacks is arduous and provides logical challenges. Menstrually related migraine provides the opportunity to investigate spontaneous attacks in a controlled manner. Thus, in the current study, we investigated spontaneous attacks of women with menstrually related migraine attacks. Exclusion criteria included pregnant or nursing women, any other primary/secondary headache disorder (except infrequent episodic tension-type headache), perimenstrual syndrome, a history of cardio- or cerebrovascular disease, substance abuse, contraindications for MRI and use of any type of daily medication (except contraceptives). Thus, none of the included participants received migraine preventive medication. Furthermore, participants with psychiatric disorders (e.g. depression, bipolar disorder and attention deficit hyperactivity disorder) were excluded based on medical history.

### Ethical approval and protocol registrations

The study was part of an umbrella protocol, which was approved by the Ethical Committee of Capital Region of Denmark (H-23054319) and registered at ClinicalTrials.gov (NCT06470958). The study was conducted in accordance with the Declaration of Helsinki with later revisions.^19^ All participants gave their written consent after receiving detailed oral and written information before study inclusion.

### Experimental design

After the first inclusion visit, participants were instructed to contact the research team at the onset of a menstrually related migraine attack. Participants arrived at the hospital during the attack and were scanned immediately upon arrival. Participants were instructed to not take acute medication before the scan. Upon arrival at the hospital, it was confirmed that criteria for menstrually related migraine attack were fulfilled, whereafter the participants underwent a high-resolution MRA with a 3 T Philips Achieva Scanner machine (Philips Medical Systems, Best, Netherlands) using a 32-element phased-array receiver head coil. Subsequently, the participants were treated with 75 mg oral rimegepant, followed by post-treatment scans at fixed timepoint at 30 and 60 min, which was the expected timepoint for maximal plasma concentration of rimegepant.^20^ Headache intensity was recorded (0 = none, 1 = mild, 2 = moderate, 3 = severe) at baseline, at 30, 60 and 120 min after rimegepant ingestion.

### Data acquisition and imaging protocols

Each imaging session began with a scout MRA that served to position and guide the subsequent three-dimensional time-of-flight (TOF) acquisition. The scout scan was obtained using a field of view (FOV) of 180 × 180 × 120 mm³ with a 120 × 120 matrix, producing an acquired voxel size of 1.5 × 1.5 × 2.4 mm³ and a reconstructed resolution of 0.20 × 0.20 × 0.35 mm³. Sequence parameters included a repetition time (TR) of 23 ms, an echo time (TE) of 3.9 ms, a 20° flip angle, SENSE acceleration enabled and two acquisition chunks, yielding a total scan duration of 1 min and 10 s.

Arterial imaging was then performed using a dedicated 3D TOF sequence. The middle cerebral artery (MCA) was imaged first with an FOV of 200 × 200 × 41 mm³ and a matrix of 800 × 571. The native voxel size was 0.25 × 0.35 × 0.70 mm³, reconstructed to 0.20 × 0.20 × 0.35 mm³. The sequence used a TR of 23 ms, a TE of 3.45 ms, an 18° flip angle, no SENSE acceleration and three acquisition chunks, for a total scan time of 9 min and 9 s. The subsequent scans of the middle meningeal artery (MMA) used identical FOV and matrix settings (200 × 200 × 41 mm³; 800 × 571) with the same acquired and reconstructed voxel dimensions. The MMA sequence applied a TR of 23 ms, a TE of 3.35 ms, an 18° flip angle, no SENSE and three chunks and had the same total duration of 9 min and 9 s.

### Imaging analysis

All angiographic datasets were processed with the LKEB-MRA vessel wall analysis software (version 6.2007), which has been validated and used extensively in migraine imaging studies.^14-16,21^ The software identifies the arterial centreline and extracts luminal contours at fixed 0.2 mm intervals along the predefined vessel segment. This procedure yielded ∼26 contour measurements for each 5 mm arterial segment.

When the automated procedure incorporated minor side branches or when contour detection was visibly inaccurate, the affected frames were reviewed and manually adjusted whenever feasible. Frames that could not be corrected were removed from the final dataset. All circumference analyses were performed by a single investigator (T.M.A.), who remained blinded to scan session and clinical status.

### Statistical analysis and sample size considerations

Demographic data are presented as means with standard deviation (SD). Arterial circumference changes are presented as means with SD.

The primary outcome of the study was a change in the circumference of the MMA and the MCA at timepoints 30 and 60 min compared to baseline. The sample size was calculated to detect at least 10% change in the circumference of the MMA, based on reports from previous studies.^14-16^ Using power = 0.90, significance level = 0.05 (two-tailed), we estimate that at least seven participants will be required.^21^ To increase confidence in our findings, we increased the sample to include at least 14 participants. Sample size in previous migraine studies investigating MMA after acute treatment ranged from 10 to 15 participants.^14-16^

We tested the difference in arterial circumferences between baseline and 30 min and between baseline and 60 min using paired samples *t*-test. Given the limited number of pre-specified comparisons, no correction for multiple comparisons was applied to avoid inflating the risk of Type II error. All analyses were conducted using IBM SPSS Statistics (version 30.0). A two-sided *P* < 0.05 was considered statistically significant.

## Results

**Figure 1:**
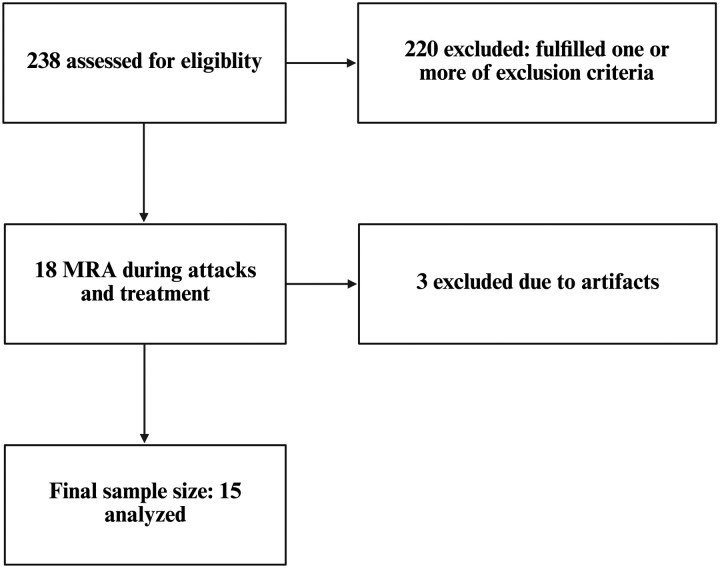
Study flow diagram. A total of 238 patients were assessed for eligibility; 220 were excluded for meeting one or more exclusion criteria. Eighteen participants underwent MRA during migraine attacks and treatment, and three were excluded due to artefacts, resulting in a final sample of 15 participants.

Between 12 January 2024 and 10 June 2025, a total of 238 women were assessed for eligibility for this study, of which 220 were excluded. Participants were excluded mainly due to comorbid conditions (18 psychiatric disorders, 1 epilepsy and 31 primary/secondary headache disorders); preventive treatments (*n* = 12); feasibility reasons, e.g. distance to the hospital (*n* = 45); not fulfilling age criteria (*n* = 15); contraindication to MRI (*n* = 13); irregular menstrual cycle (*n* = 10); lost to follow-up (*n* = 53) pregnancy/breastfeeding (*n* = 14); or fulfilling other exclusion criteria (*n* = 8). Eighteen participants were scanned before and at fixed timepoints (30 and 60 min) after treatment with 75 mg oral rimegepant. Two participants were subsequently excluded from analysis due to motion artefacts on the acquired images. Data for one participant were missing at timepoint 60 min because of motion artefacts. Thus, a total of 15 participants were included in the final analysis (Fig. 1).

### Demographic characteristics

The mean age of the participants was 29.7 years ± 5.3 SD (range 19–39 years), mean disease duration 11.3 years ± 6.5 SD, mean monthly headache days 4.3 ± 2.3 SD and mean monthly migraine days 3.6 days ± 2.1 SD. None of the participants fulfilled criteria for chronic migraine, and none of the participants had ever had an episode of migraine aura. The median time for headache onset until MRA scan was 8 h. At baseline, 11 of 15 participants (73%) had bilateral headache (see Table 1). All participants were scanned during menstrually related migraine attacks and were treated with rimegepant. At timepoint 2 h, one (7%) of 15 participants had pain freedom, while additional three (20%) participants had pain relief.

**Table 1:** Demographic data and clinical characteristics at baseline (during attacks)

	All *N* = 15
Mean age (SD)	29.7 (5.3)
MMD (SD)	3.6 (2.1)
MHD (SD)	4.3 (2.3)
Mean disease duration years (SD)	11.3 (6.5)
Phonophobia (***N***)	75.0% (12)
Bilateral headache (***N***)	73% (11)
Unilateral headache Left side (%) Right side (%)	27% (4) 7% (1) 20% (3)
Nausea (***N***)	62.5% (9)
Photophobia (***N***)	87.5% (13)
Phonophobia (***N***)	75% (11)
Hours from onset until scan, median (IQR)	8 (3.3–12.0)

*N*, number; SD, standard deviation.

### Side-to-side differences of arterial circumference at baseline

We found no baseline differences between the right-sided and left-sided MMA (*P* = 0.509) or MCA (*P* = 0.715). Therefore, the means of the right and left MMA and MCA circumference were used for further comparison (see Table 2).

**Table 2:** Mean baseline values of MCA and MMA

	Left MCA (SD)	Left MMA (SD)	Right MCA (SD)	Right MMA (SD)
Mean (SD)	8.2 mm (1.2)	4.3 mm (0.8)	8.1 mm (0.9)	4.4 mm (0.9)

### Circumference changes after rimegepant

**Figure 2:**
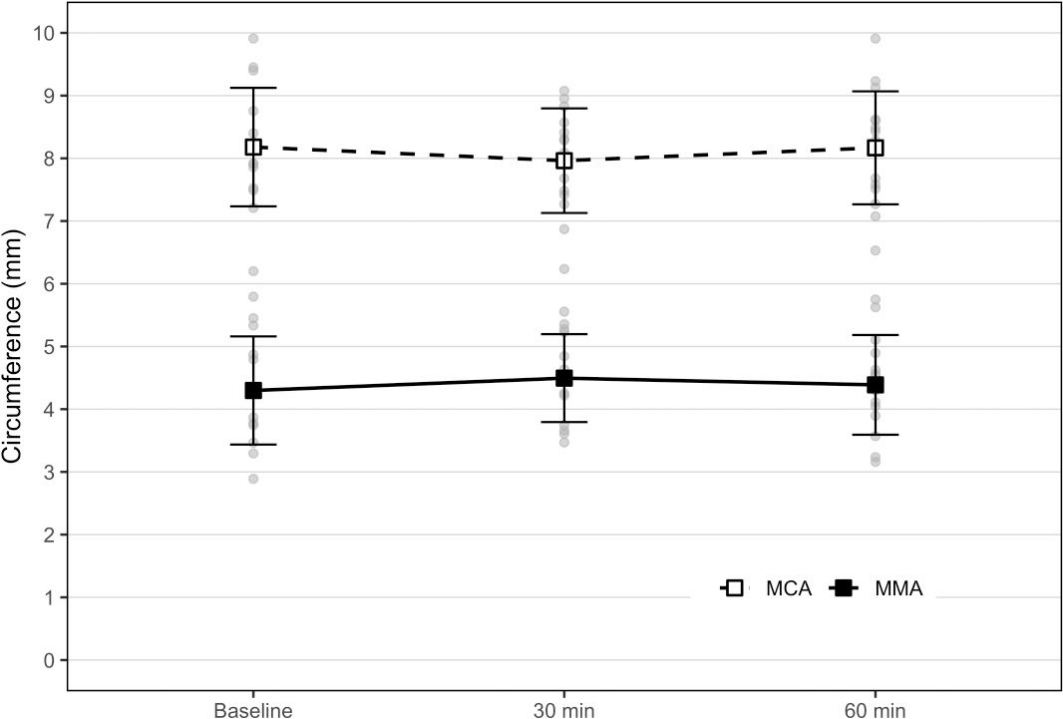
Changes in circumference. Absolute values of the MCA and the MMA before and after 75 mg oral rimegepant ingestion. The punctured line represents the MCA, while the full line represents the MMA. Error bars represent the SD. The circumference of the MCA was 8.12 ± 0.93 SD at baseline, 8.02 mm ±0.84 SD at 30 min and 8.15 mm ±0.90 SD at 60 min. The circumference of the MMA was 4.30 ± 0.83 SD at baseline, 4.47 mm ±0.68 SD at 30 min and 4.35 mm ±0.78 SD at 60 min. The per cent circumference change between baseline and 30 min was −1.1% (95% CI, −4.2–2.1) for the MCA and 5.1% (95% CI, −0.5–10.7) for the MMA. The per cent circumference change between baseline and 60 min was 0.5% (95% CI, −3.6–4.6) for the MCA and 2.0% (95% CI, −4.3–8.4) for the MMA.

We found no difference in the MCA circumference between baseline (8.13 ± 0.93 mm) and after [30 min, 8.02 ± 0.84 mm (*P* = 0.404); 60 min, 8.15 ± 0.90 mm (*P* = 0.918)] treatment. There was also no difference in the MMA circumference between baseline (4.30 ± 0.83 mm) and after [30 min, 4.47 ± 0.68 mm (*P* = 0.084); 60 min, 4.35 ± 0.78 mm (*P* = 0.688)]. Additionally, there was no circumference change between timepoints 30 and 60 min for MCA (*P* = 0.471) or MMA (*P* = 0.284) (Fig. 2).

## Discussion

This study is the first to investigate the *in vivo* vascular effects of rimegepant during menstrually related migraine attacks using high-resolution MRA. Our main finding was that oral administration of 75 mg rimegepant did not induce any changes in the circumference of either the MCA or the MMA within 60 min after intake, despite the expected onset of pharmacological and clinical action. Our results demonstrate that rimegepant lacks both direct and indirect vasoconstrictive effects on cephalic arteries during migraine attacks.

Although acute or short-lasting vasodilation is not inherently painful in individuals with migraine,^22^ MRA studies conducted over the past decades have consistently demonstrated ipsilateral arterial dilation during migraine attacks.^14-16^ This ipsilateral dilation is most likely a consequence of, and a surrogate marker for, activation of trigeminal perivascular nerve fibres and the local release of several vasoactive substances, which includes CGRP.^23^

The CGRP antagonists lack intrinsic vasoconstrictive properties. Yet concerns have been raised that counteracting the vasodilatory effects of CGRP with the anti-CGRP treatment might lead to arterial constriction. In the present study, the effect of rimegepant was investigated during migraine attacks, when perivascular CGRP concentrations may presumably beat the highest level. Under such conditions, if CGRP receptor blockade was to induce a so-called reciprocal vasoconstriction, a measurable reduction in arterial diameter would be expected. Yet this vasoconstriction was not observed during the study period of the present study. In support, one study demonstrated that erenumab, a CGRP receptor antibody used for migraine prevention, did not alter cerebral vasomotor reactivity or endothelial function in patients with migraine without aura up to 4 months of treatment.^24^ Another possible explanation is that CGRP antagonism works by preventing CGRP signalling that sustains the migraine attack, rather than by reversing activation of already engaged CGRP receptors. Another co-reason may be that other vasoactive peptides, such as pituitary adenylate cyclase-activating polypeptide,^25^ might also have been present, maintaining arterial dilation despite CGRP receptor antagonism. These mechanisms could explain the relatively poor acute antimigraine effect of rimegepant (7% pain freedom and 20% pain relief) reported in the present study, where treatment was not blinded. An exploratory analysis in the present study showed no indication of a correlation between efficacy and vasoconstriction (data not shown), suggesting that rimegepant’s antimigraine effect may be mediated via CGRP-dependent modulation of trigeminovascular signalling pathways rather than via vasoconstriction.^3,26,27^

Rimegepant is a highly potent CGRP receptor antagonist with strong binding affinity. In *in vitro* binding assays, the inhibition constant (Ki) is ∼0.027 nM, indicating very high receptor affinity.^28^ Functional studies show that the concentration required to inhibit 50% of CGRP-induced cAMP production (IC₅₀) is about 0.14 nM.^28^ After a single oral dose of 75 mg rimegepant, the maximal plasma concentration (*C*_max_) is ∼1614 nM, reached at around 1 h (*T*_max_).^20^ These values highlight the drug’s high potency, as therapeutic plasma concentrations after oral dosing exceed the IC₅₀ by several orders of magnitude. These values also indicate that substantial pharmacological activity is already present at 60 min, which was our study window, and most likely already at 30 min. Taking together, the study window until 60 min was sufficient to detect pharmacological effects of rimegepant in the present study. One participant achieved pain freedom and three reported pain relief at 2 h after rimegepant. However, a potential full therapeutic effect of rimegepant may not have been captured within our study window. This observation suggests that vascular and clinical effects may occur on different timescales.

The MRA technique employed in this study has been previously validated^21^ and used in several investigations examining the effects of sumatriptan on arterial circumferences.^14-16^ Given that the MMA is the smallest vessel assessed, concerns might arise regarding the ability to detect vasoconstriction. In Amin *et al*.,^14^ we demonstrated that in 15 female participants with migraine without aura scanned during spontaneous migraine attacks and again 30 min after a 6 mg subcutaneous sumatriptan injection, there was a significant constriction of the MMA on the pain side (from 4.31 ± 0.66 mm to 3.56 ± 0.55 mm; −17.0%) and the non-pain side (from 4.24 ± 0.62 mm to 3.56 ± 0.44 mm; −15.2%). In contrast, the circumference of the MCA remained unchanged (pain side: from 10.04 ± 1.42 mm to 9.76 ± 1.45 mm; −2.5%). Similar findings were reported in Asghar *et al.*,^15^ where 15 participants with CGRP-induced migraine attacks without aura were scanned before and 15 min after 6 mg subcutaneous sumatriptan injection. Furthermore, in the present study, we applied identical acquired voxel resolution and reconstructed resolution to those used by Asghar *et al.*^15^ and Amin *et al*.^14^ We therefore have strong confidence in our findings that, in contrast to sumatriptan, rimegepant does not induce constriction of either cerebral or meningeal arteries. However, we cannot exclude subtle vascular changes based on our sample size. A *post hoc* power analysis showed that in our sample of 14 patients, the minimum detectable change in MMA circumference would be 6.6%, which is not likely considered a clinically meaningful difference.

The present study design assessed the acute vasoconstrictor effect of rimegepant on potentially dilated MCA and MMA in women with menstrually related migraine attacks. Given that perivascular CGRP concentrations are highest during ongoing attacks, examining vasoconstrictor effect in the interictal phase would most likely also show no vasoconstrictor effect of rimegepant. Moreover, the effect of long-term use of rimegepant or use in individuals with known increased cerebrovascular risk was not investigated. In individuals with cerebrovascular dysfunction, both CGRP and CGRP antagonisms may probably be even less effective. This design ensured that vascular measurements were obtained during the early phase of the migraine attack, when pathophysiological changes are expected to be most pronounced. Finally, the sample size was calculated to visualize vasoconstriction and not treatment efficacy, which would require much higher number of participants. Only four patients experienced efficacy of rimegepant limiting subgroup analysis.

In conclusion, these results indicate that rimegepant does not cause measurable constriction of either the MCA, representing the intracerebral circulation, or the MMA, representing extracerebral vasculature. This underscores the use of rimegepant as a safe therapeutic alternative for patients with cerebrovascular risk, where triptans remain contraindicated.^29,30^ In addition, these findings may reinforce the concept that migraine treatment does not require vasoconstriction,^17^ which has been a central limitation of triptan therapy for decades.

## Conclusion

Rimegepant did not induce measurable vasoconstriction of the middle cerebral or middle meningeal arteries during spontaneous migraine attacks. These findings support its vascular safety and reinforce that effective migraine treatment can be achieved without vasoconstriction, challenging a key limitation of triptan-based therapies.

## Data Availability

Upon reasonable request, the corresponding author will provide the necessary data and materials to interested researchers for the purpose of academic scrutiny, reproducibility and further scientific investigation.
